# Seeing the Small to Prevent the Big: The Role of Cerebral Microvessels in Ischemic Stroke Etiology and Prevention

**DOI:** 10.1155/srat/8417271

**Published:** 2026-09-15

**Authors:** Kin Tung Jonathan Chan

**Affiliations:** ^1^ College of Medicine and Health, University of Birmingham, Birmingham, UK, birmingham.ac.uk

**Keywords:** cerebral small vessel disease, diagnostic imaging, microvessels, preventative medicine, stroke

## Abstract

Despite decades of progress in its management, ischemic stroke remains a leading cause of death and disability worldwide, with incidence rising in younger populations. Although traditional stroke risk assessment focuses on macrovascular pathology, the cerebral microcirculation plays an important but less‐recognized role. This review synthesizes evidence on microvascular involvement in the pathogenesis of ischemic stroke and evaluates existing and emerging methods for microvascular assessment of ischemic stroke risk (MvASR). Epidemiological studies show that cerebral small vessel disease as measured by MRI biomarkers significantly increase stroke risk, with risk rising alongside lesion progression. Microemboli animal models reveal that even asymptomatic microemboli can disrupt the blood‐brain barrier and trigger inflammatory changes that may predispose to stroke. Postischemia models demonstrate that no‐reflow and prolonged microvascular inflammation compromise patency, potentially increasing recurrent stroke risk. Current MvASR modalities can be classified into direct imaging (cerebral angiography, retinal photography), indirect imaging (MRI, transcranial Doppler), and nonimaging approaches (e.g., genetic testing for CADASIL). Although more studies demonstrating clinical utility are needed, the accumulating pathophysiological and epidemiological evidence suggests that MvASR will become increasingly relevant to stroke prediction and prevention.

## 1. Introduction

Despite improvements to stroke management and care over the past few decades, stroke remains a leading cause of death worldwide. For example, stroke is the 4th leading cause of death in the United Kingdom, and although stroke‐attributed deaths there have decreased by ~45% in 2002–2018, stroke incidence did not improve contemporaneously [1, 2]. In fact, stroke incidence increased in people aged < 55 over that period, diverging from the trend in older people. A recent meta‐analysis demonstrated similar divergence in 19 other high‐income countries [3].

As most strokes are first‐time strokes, and because strokes carry great morbidity, stroke prevention is important [4]. Stroke has traditionally been thought of as having a largely macrovessel pathology, and stroke risk factors such as carotid artery disease, atherosclerosis, coronary artery disease, tend to reflect this paradigm [5]. The role of microvessels is comparatively less widely known, even though the microvascular‐specific phenomena of no‐reflow and microemboli have important ramifications on stroke prediction, prevention, and prognosis. For the purposes of this review, we define microvascular assessment of stroke risk (MvASR) as the evaluation of microvascular structure and function (e.g., microemboli burden, no‐reflow, vasoreactivity) using imaging or biomarker‐based approaches, with the goal of stratifying risk of initial and recurrent stroke. Emerging microvascular assessment methods can make important contributions to this domain.

The aim of this narrative review is twofold: to present evidence on the microcirculatory aspects of stroke etiology and to review existing microvascular investigations that may have a role in predicting stroke. As most strokes are ischemic (~87% in the United States and ~65% of strokes globally are ischemic), this review largely focuses on ischemic strokes [6, 7].

## 2. Methods

The literature for this narrative review was identified through searches of PubMed/MEDLINE, supplemented by snowballing. Keywords and combinations thereof included those related to stroke and microvessels (“stroke,” “microvascular,” “microcirculation,” “microvessel”), cerebral small vessel disease (CSVD) (“CSVD,” “cerebral small vessel disease,” “white matter hyperintensit∗,” “cerebral microbleed”), pathological phenomena related to microvessels (“microemboli,” “no‐reflow,” “microvascular inflammation”), animal models (“model,” “animal model,” “mouse model”), and imaging or other assessment modalities (“imaging,” “MRI,” “CT,” “ultrasound,” “assessment,” “biomarker,” “prediction”). We included English‐language articles published before May 2026. Studies were included if they investigated the relationship between microvascular biomarkers and stroke risk, studied in vivo models of stroke related to the microcirculation, or were concerned with the viability or effectiveness of microvascular investigations to assess stroke risk. In vitro models of stroke and case reports were excluded.

## 3. Microcirculation in the Etiology of Stroke

The concept of “microvessel” still lacks a universally agreed‐upon definition. Typical definitions use vessel diameter cutoffs; commonly cited figures say 100–200 *μ*m [8, 9]. However, microvasculature of different organs includes vessels of much larger size [10, 11]. Indeed, one survey of 13 neuropathology centers in 10 countries showed < 50% agreement on a definition of “small vessel” [12]. Regardless of definition, disease or occlusion of the cerebral microvasculature is often asymptomatic and is still often found in those with low cardiovascular risk factors [13, 14]. Therefore, greater understanding of cerebral microvascular dysfunction in stroke may translate into new diagnostic methods if detectable microvascular changes are proven to predict stroke occurrence.

As it turns out, the microcirculation is involved at multiple stages of stroke. Not only does microcirculation play a role in etiology, it is also a key player in pathogenesis. Studies of pathogenesis have primarily focused on microvascular endothelium‐leukocyte/platelet dynamics in the stroke process [15, 16], and despite difficulties in translating such knowledge into successful drug trials, this line of investigation may be starting to bear fruit [17–19]. However, as this article focuses on methods to assess risk of stroke, we restrict this section′s attention to microcirculatory etiology of stroke.

### 3.1. Epidemiological Evidence

CSVD refers to a heterogenous group of diseases affecting the cerebral microcirculation [20]. Roughly, CSVD is stratified by the implicated vascular limb (arterial/venous) and pathophysiology (e.g., inflammatory), as these can alter the clinical picture. In Type 1 CSVD (arteriolosclerosis), for example, fibro‐hyaline deposition into arteriolar lumina thickens vessel walls, increasing risk of ischemic stroke (where bloodflow is obstructed in a vessel); paradoxically, it also weakens vessel walls, increasing risk of rupture and hemorrhagic stroke (bloodflow is diverted into extravascular space) [20, 21]..

Epidemiological studies show that CSVD is associated with increased risk of stroke, with risk increasing with CSVD severity [22, 23]. CSVD therefore suggests a strong association between microcirculatory lesions and stroke, but causation remains controversial since established risk factors such as hypertension and diabetes mellitus (DM) commonly occur alongside CSVD [5, 20]. Moreover, controlling such risk factors tends to limit the progression of CSVD, raising the possibility that microcirculatory abnormalities are themselves not an independent risk factor, but instead a common pathology dependent on other risk factors [24, 25].

However, studies of MRI biomarkers in relation to cerebrovascular injury suggest that microcirculatory abnormalities form an independent risk for stroke. The Standards for Reporting Vascular Changes on Neuroimaging (STRIVE) guideline considers various MRI biomarkers that represent direct evidence of cerebral microvascular injury, of which the most well‐established (since STRIVE‐1) include white matter hyperintensities (WMHs) and lacunes of presumed vascular origin, recent small subcortical infarcts, perivascular spaces (PVSs), and cerebral microbleeds (CMBs) [26, 27]. The exact mechanisms linking these MRI changes with microvascular dysfunction remain uncertain (see Table 1), but counting and grading of these lesions have become a cornerstone of CSVD diagnosis [20, 28].

**Table 1:** Theorized pathophysiology underlying key MRI biomarkers of CSVD [20, 26, 27].

MRI biomarker of CSVD	Summary of theorized pathophysiology
White matter hyperintensities (WMHs)	Degeneration of myelinated fibers following chronic hypoperfusion due to microvascular narrowing
Recent small subcortical infarcts and lacunes	Evidence of infarction in the territory of a small perforating arteriole at different chronological stages
Perivascular spaces (PVSs)	Impaired clearance of interstitial fluid around a vessel due to multiple causes, for example, vessel wall damage, hypertension
Cerebral microbleeds (CMBs)	Microvessel rupture due to chronic vessel wall damage, or amyloid angiopathy

The most recent meta‐analysis of longitudinal studies investigating the clinical significance of CSVD‐related MRI biomarkers in community‐dwelling older adults found that WMHs, MRI‐defined covert brain infarcts (mostly subocortical), and CMBs were each associated with a significant increase in stroke risk. WMHs conferred the greatest risk: extensive WMH burden in the general population conferred a significant increase in ischemic stroke risk (hazard ratio 2.39, 95% CI 1.65–3.47) [23]. Restricting to studies of “high‐risk” populations (e.g., those with previous stroke, mild cognitive impairment, etc.), the risk was greater (hazard ratio 2.78, 95% CI 1.43–5.40). A caveat in interpreting the pooled hazard ratios is that most, but not all included studies adjusted for hypertension and other vascular risk factors. Furthermore, although too few eligible studies on PVSs could be found, the meta‐analysis noted that the current literature showed a weaker association between PVSs and ischemic stroke risk after adjusting for vascular risk factors, which may be due to PVSs being associated with other nonvascular etiologies (e.g., age, genetics) [20, 26].

As there was significant heterogeneity in the reporting of biomarker severity, the meta‐analysis did not systematically investigate biomarker progression and stroke risk. However, recent analysis of the ARIC longitudinal study (general population, *n* = 907 for those with MRI, aged 45–64 when study began) found significant positive correlation between progression in MRI biomarkers and stroke risk (presence of lacune or WMH grade ≥ 2, hazard ratio 2.16, 95% CI 1.10–4.24) that was also “dose‐dependent” (lacune with WMH grade ≥ 2, hazard ratio 4.36, 95% CI 1.73–11.0) [29].

If biomarker progression increased stroke risk, and biomarker regression decreased this risk, the case for microvascular changes (by biomarker severity) directly modulating stroke risk would be even greater. Physiological interpretation of reversal is not readily clear in all biomarkers, such as in the case of CMBs. However, in the case of WMHs, regression could suggest improved perfusion due to less microvessel stenosis. Thus far, only one longitudinal study has explored the WMH volume‐stroke risk relationship, and due to its population of stroke patients, its findings are of limited value for our case [30]. Moreover, while this study showed that regression of WMH (normalized by intracranial volume) was associated with significant reductions in stroke re‐occurrence, WMH regression was also strongly associated with blood pressure reductions, which does not necessarily support either side of the argument that microcirculatory changes independently modulate stroke risk.

### 3.2. Animal Models

Although epidemiology suggests existence of mechanisms, animal models can actually reveal them. We restrict ourselves to models that are most pertinent for discussion in Section 4 below. We separate these models into three types: microemboli models (which better model the etiology of first strokes), postischemia models (better for second strokes), and models of endothelial senescence.

#### 3.2.1. Microemboli Animal Models

Approximately 25% of strokes are pre‐empted by a transient ischemic attack (TIA) [31]. TIAs are characterized by focal neurological deficits, typically lasting < 1 h, and lack visible lesions on standard neuroimaging [31]. Because TIA are most commonly transient embolic events, it is reasonable to conjecture that circulating microemboli (i.e., tiny emboli that can potentially cause embolisms in the microvasculature) at some low level could be an asymptomatic “warning shot” for TIA or stroke, and a greater understanding of the relationship between microemboli levels and stroke symptoms may lead to microemboli becoming a useful biomarker for stroke risk [32]. Although this conjecture sounds like a truism, interpretation of microemboli “signals” could potentially be distorted by the body′s endogenous thrombolytic mechanisms, like tissue‐type plasminogen activator (tPA) [33, 34]. However, these endogenous mechanisms may not be so significant in the brain, as many studies establish an “anti‐hemorrhage” bias in cerebral vasculature relative to systemic vasculature, which manifests as favoring coagulation (e.g., upregulating tissue factor) over anticoagulation (e.g., downregulating tPA) [34].

Microemboli models stem from a line of animal models intended to validate thrombolytic or neuroprotective therapies in stroke; other models include filament occlusion, ligature, coagulation, and emboli [35–39]. Most early microemboli models were designed to reliably induce stroke symptoms, not model the asymptomatic range of microemboli [40–42]. Thus, although microemboli of certain large size and number induced stroke, it was probably at levels beyond relevance to stroke risk assessment. Later, Atochin et al. developed a microemboli model based on fluorescent fibrin “microclots,” and they were likely first to systematically measure histological and neurological consequences of different dosages of microemboli [38]. The microclots used were much smaller than the “microspheres” of previous microemboli studies (Table 2) [37, 38, 41], and were confirmed to have reached the microcirculation. Using a 5‐point scale, they demonstrated a significant, dose‐dependent relationship between microemboli size and neurological deficit, with the “large” dose causing obvious stroke symptoms, and the “small” dose asymptomatic [38]. In other words, variations in microemboli levels cause different severities of stroke, and there is a small but nonnegligible level that is asymptomatic.

**Table 2:** Table of microsphere statistics across studies cited in the present article, listed in ascending order of microemboli aggregate volume, that is, the product of mean microsphere diameter and number of spheres used. However, note that aggregate volume is a crude measurement of microemboli dosage as it assumes biological equivalence of many small emboli against few large emboli of matched volume, whereas the likely affected vascular territories and resulting pathology would differ.

Mean microsphere diameter (*μ*m)	Number of spheres	Aggregate volume (nanoliter)	Reference	Study year	Type	Remarks
12.50	2500	2.557	Lam et al. [43]	2010	Microclot	
2.75	400,000	4.356	Atochin et al. [38]	2004	Microclot	“Small,” found to be asymptomatic
2.75	800,000	8.711	Atochin et al. [38]	2004	Microclot	“Moderate”
2.75	1,400,000	15.245	Atochin et al. [38]	2004	Microclot	“Large”
15.00	15,000	26.507	Georgakopoulou et al. [44]	2023	Microsphere (polystyrene)	“Group 15” (small)
48.00	600	34.744	Takeo et al. [41]	1989	Cholesterol crystal	
47.50	680	38.158	Miyake et al. [42]	1989	Microsphere (polystyrene)	
25.00	5500	44.997	Georgakopoulou et al. [44]	2023	Microsphere (polystyrene)	“Group 25” (medium)
15.00	27,500	48.597	van der Wijk et al. [45]	2019	Cholesterol crystal	
50.00	1000	65.450	Mayzel‐Oreg et al. [46]	2004	Microsphere (polyethylene)	
50.00	1000	65.450	Georgakopoulou et al. [44]	2023	Microsphere (polystyrene)	“Group 50” (large)
40.00	3500	117.286	Wang et al. [47]	2012	Cholesterol crystal	
17.47	31,125	130.082	van der Wijk et al. [45]	2020	Microsphere (polystyrene)	Wide distribution of microsphere diameters
50.00	2000	130.900	Wiernsperger et al. [40]	1984	Microsphere (carbonized plastic)	
14.00	?	Unknown	Lam et al. [43]	2010	Cholesterol crystal	Not enough information to determine dose

A caveat of using these microemboli studies to understand the microemboli‐stroke relationship is that most of their time‐horizons were short (typically 1–2 days) [38, 40, 42, 48, 46]. Some later studies used longer time‐horizons (7–28 days), but very few used microemboli dosage similar to the “low” dose in Atochin et al. that mimics asymptomatic microemboli levels (Table 2).

Using in vivo two‐photon microscopy in similar microemboli models, Lam et al. discovered a new process whereby microemboli were translocated across cerebral endothelium (“angiophagy”). Angiophagy was later discovered in systemic endothelium as well [43, 49], but given cerebral vasculature′s inhibition of anticoagulation, angiophagy should play a greater role in the brain [34]. Notably, Lam et al.′s microemboli studies used a microemboli dosage (in terms of aggregate volume, see Table 2) below the “low” dosage of Atochin et al. [38]. This suggests that chronic, but low, asymptomatic levels of microemboli may increase stroke risk via the angiophagy mechanism. At high levels of circulating microemboli, “overloading” of the angiophagy mechanism could lead to interstitial expansion and luminal occlusion. However, at even low levels of circulating microemboli, chronic activation of angiophagy may lead to long‐term increases in membrane permeability, which can have downstream inflammatory changes predisposing to stroke. Indeed, some level of blood‐brain barrier (BBB) disruption is necessary to mediate angiophagy [43, 45, 50]. Van der Wijk et al. used IgG‐immunofluorescent staining to measure the spatio‐temporal dynamics of BBB disruption in angiophagy, and found that within 24 h, microsphere extravasation caused “profound” vascular leakage of endogenous IgG, indicating BBB disruption [45]. This disruption was temporary, lasting 3–7 days. It is thus likely that BBB disruption can be exacerbated by low, chronic microemboli presence. Although IgG‐immunohistochemistry in these experiments intended to detect BBB disruption, we speculate that IgG accumulation in PVSs increases risk of platelet activation through the generation of perivascular immune complexes, which can recruit inflammatory cells and induce endothelial activation [51]. Georgakopoulou et al., using varying doses of microemboli, found IgG leakage was more widespread at lower microsphere diameters [44]. Taken together, the studies above suggest that long‐term, asymptomatic microemboli exposure increases stroke risk, but more studies using lower dosages are needed.

#### 3.2.2. Postischemia Animal Models

Stroke history is an important risk factor for recurrent stroke: ~26% of stroke survivors have a second stroke within 5 years of their first [52]. This risk is multifactorial, with emphasis on original stroke etiology and post‐stroke management [53]. In other words, the main reason stroke appears to be a risk factor for another is because the first stroke is a proxy of an underlying pathology (e.g., atrial fibrillation). However, post‐stroke models of the microvasculature show that microcirculatory changes themselves play a role in increasing risk of second stroke, regardless of etiology.

Survival of ischemic stroke hangs on reperfusing ischemic regions [53]. However, reperfusion is not benign. Intravital microscopy experiments show that, upon reperfusion of murine stroke models, an inflammatory response recruits leukocytes and upregulates adhesion molecules such as L‐selectin and intercellular adhesion molecule 1 (ICAM‐1) in cerebral endothelium, risking occlusion (leukocytes being larger and less deformable than erythrocytes) [15, 54]. A thrombogenic response lags behind, with platelet rolling and adhesion in cerebral venules trailing post‐stroke inflammation by hours [15, 55]. Longer experiments (up to 28 days) show subsequent immunosuppression, probably due to leukocyte migration to the brain, which may be neuroprotective by mitigating development of anticentral nervous system (CNS) antibodies [56, 57]. This prolonged period of post‐stroke immunosuppression has been observed in humans, and can lead to long‐term persistence (months–years) of inflammatory infiltrate in cerebral microvessels [56, 58]. By remodeling the microvasculature and making it narrower, postischemic inflammation jeopardizes microvascular patency.

Reperfusion is also a matter of degree: Many ischemic stroke patients encounter “no‐reflow”, where successful recanalization (flow restoration at the primary occlusive site) only leads to partial reperfusion (flow restoration at distal branches); the reduced perfusion tends to be long‐lasting [59]. No‐reflow occurs in other vascular beds [10, 59, 60], but in the brain it is especially formidable due to the endogenous bias towards coagulation [34], as well as the magnified consequences of hemorrhage in thrombolysis/thrombectomy [53]. Losing collateral cerebral microvessels to no‐reflow probably increases risk of second stroke [61].

Intravascular mechanisms of no‐reflow were investigated first, such as thrombus formation within microvessels. In a baboon model of stroke, radioisotope‐labeled platelets were found to deposit in microvessels shortly after recanalization [62]. Immunohistochemical and electron‐microscopy studies of the same model detected significant upregulation of P‐selectin and ICAM‐1 expression in brain‐ischemic regions [63], as well as platelet degranulation [64]. Platelet activation has been attributed to subendothelial collagen exposure following ischemic/reperfusion injury. Microemboli can cause no‐reflow in microvessels too: using two‐photon microscopy, one group observed neutrophils stalling in cerebral capillaries following recanalization in a mouse middle cerebral artery occlusion (MCAO) model [65]. They further demonstrated the importance of neutrophils by showing depletion of neutrophils with anti‐Ly6G antibody administration before MCAO significantly improved cerebral bloodflow (CBF) 120 min post‐stroke (70% of baseline CBF with anti‐Ly6G‐Abs vs. 54% without, *p* < 0.01).

Extravascular mechanisms are increasingly recognized in no‐reflow. Following ischemia, astrocytic end feet enwrapping microvessels become edematous, constricting them [60]. Astrocytic edema likely has several causes but much focus has been on AQP‐4, an aquaporin almost exclusively expressed by astrocytes (among CNS cells) [66]. Brains of AQP4(‐/‐) mice were found to have significantly less edema following MCAO‐induced ischemia, as well as better neurological outcomes [67]. A MCAO model similarly found that inhibiting an upstream MAP‐kinase (p38) of AQP‐4 significantly reduced MCAO‐induced edema [68]. Interestingly, AQP‐4 regulation accords with the brain′s “bias” against hemorrhage mentioned above, as AQP‐4(‐/‐) mice fare worse in models of cerebral vasogenic edema (common consequence of hemorrhagic stroke) [66]. Microvascular pericytes, which are contractile thanks to *α*‐smooth muscle actin, may be another important extravascular factor; following infarction, pericytes may stay contracted as loss of ATP generation increases pericyte cytoplasmic intracellular calcium concentration [60]. A MCAO model demonstrated pericyte contraction lasting up to 24 h postischemia [69]. However, pericytes′ contractility is a controversial topic [70, 71], and a recent MCAO study compared different microvessel segments of varying pericyte densities and concluded that pericytes, whether they really contract or not, unlikely play a significant role in microvessel constriction postischemia [70].

### 3.3. Endothelial Senescence as Driver of Microvascular Dysfunction

Advancing age is an important nonmodifiable risk factor for stroke [6], and although age may reflect the accumulation of comorbidities over time (e.g., diabetes, hypertension), endothelial cellular senescence provides a microvascular mechanism for aging itself as an independent risk factor for stroke. Additionally, senescence may exacerbate the damage caused by ischemia and microembolic burden.

Senescent endothelial cells (ECs) exhibit hallmark features including irreversible cell cycle arrest, morphological changes, and a proinflammatory senescence‐associated secretory phenotype (SASP) partly characterized by increased secretion of proinflammatory cytokines and matrix metalloproteinases [72]. This chronic proinflammatory environment induces maladaptive remodeling and BBB disruption, predisposing to stroke [73]. Senescent ECs also have increased secretion of plasminogen activator inhibitor‐1 (PAI‐1), which may increase thrombotic risk as well as risk of no‐reflow states by reducing the effectiveness of thrombolytic therapy in stroke. Furthermore, senescent ECs exhibit impaired migration, reducing their capacity for angiogenesis and further increasing the risk of no‐reflow [72].

In mouse models, this senescence‐induced microvascular dysfunction was shown to cause vascular cognitive impairment, which in humans is a common consequence of CSVD and partly represents accumulated infarct burden [28, 74]. Recent work using a single‐cell transcriptomics approach on the p16‐3MR murine model of cellular senescence showed that cerebral ECs were particularly sensitive to chronological aging compared with other brain cell types [73]. When the authors used senolytic methods to eliminate the senescent cells, not only did histological evidence of microvascular dysfunction (including BBB disruption) recede, but cognitive performance was also improved, suggesting a potential therapy for CSVD and hence a new means of reducing stroke risk.

## 4. MvASR: Existing Methods

**Figure 1:**
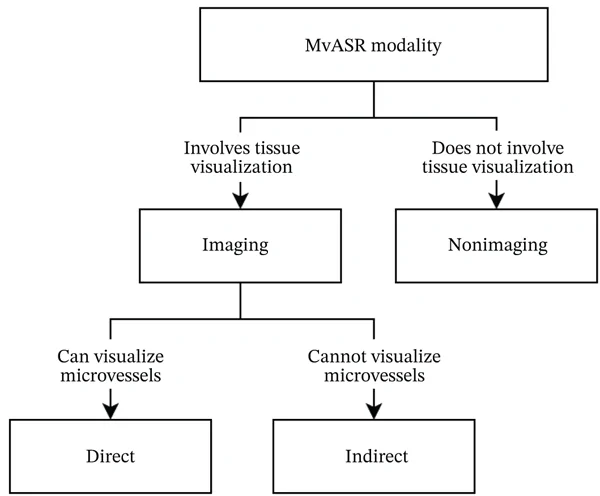
Classification of MvASR investigations used for this section. Microvessels were arbitrarily defined as being < 200 μm wide for this classification.

The growing repertoire of microvascular changes associated with stroke suggests that clinicians may benefit from directly assessing these changes when evaluating stroke risk. In recent years, an emerging collection of techniques for MvASR has benefited greatly from concurrent innovations in imaging and computing. These methods are now numerous enough to merit a classification scheme (Figure 1). Imaging refers to any method of assessment where visual representation of tissues is central. Nonimaging methods include genetic testing and blood tests. Imaging methods are further classified as direct or indirect, depending on spatial resolution—whether it can visualize microvessels directly or not (for purposes of this section, microvessel diameter is < 200 *μ*m).

The distinction between direct and indirect imaging MvASR methods is not concrete and changes with continued technological advancement. However, it highlights the fact that it is still possible to assess microvascular structure and function without “seeing” them. Furthermore, though microvessels are defined by their smallness, temporal resolution (how quickly images can be captured) and tissue depth are important considerations too (Table 3) [75]. Additionally, different imaging modalities are sensitive to different structures, so that no method will completely displace another in MvASR. The MvASR techniques covered in this section are linked to their respective clinical scenarios in Table 4 below.

**Table 3:** Comparison of best spatial and temporal resolutions of different imaging modalities mentioned in this section; cited values for retinal photography [76], DSA [77], 3D/4D‐DSA [78], ultrahigh resolution CT [79], MRI [80], and ultrasound [75, 81] are approximate best figures that ultimately depend on specific vendors. Greatest spatial resolution is typically achieved at the expense of temporal resolution and vice‐versa, so that the above spatio‐temporal resolution combinations are unlikely to be attainable in practice.

Modality	Spatial resolution	Temporal resolution	Clinical readiness
Retinal photography	~7 *μ*m	Milliseconds	Emerging
Digital subtraction angiography (DSA)	50 *μ*m	Seconds	Clinically established
3D‐DSA/4D‐DSA	150 *μ*m	Seconds	Emerging
Ultrahigh resolution CT	150 *μ*m	Minutes	Experimental
MRI	300 *μ*m (7‐Tesla)	Seconds	Clinically established (CSVD biomarkers, perfusion‐weighted imaging) Emerging (fractal dimension)
Ultrasound	50 *μ*m	Milliseconds	Clinically established (transcranial Doppler, ultrasound microvascular flow imaging)

**Table 4:** Summary of MvASR techniques mentioned in this section alongside the microvascular process they are intended to detect and how the process is theorized to contribute to increased risk of stroke (initial or recurrent). More details are found in the sections discussing each technique.

Microvascular process	Mechanistic contribution to stroke risk	MvASR modalities
No‐reflow	Impaired reperfusion, infarct expansion	Digital subtraction angiography (DSA), 4D‐DSA, MRI‐perfusion weighted imaging
Impaired vasoreactivity	Reduced autoregulation, chronic vessel wall damage	Retinal photography (fractal analysis of retinal vessels), MRI (fractal dimension of cerebral vessels)
Microemboli	Acute occlusion, chronic hyperperfusion, BBB disruption	Transcranial Doppler, ultrasound microvascular flow imaging (MVFI)
Genetic CSVD risk	Various mechanisms	Genetic testing (e.g., *NOTCH3* mutation for CADASIL)
Neovascularization of peripheral atherosclerotic plaques	Plaque embolization	Ultrasound MVFI

This MvASR classification leaves out invasive technologies; invasive means (e.g., laser speckle contrast imaging) have been used in medical practice to assess microvasculature but are less likely to see widespread use to assess stroke risk [82]. Similarly, although intravital techniques involving microscopy or functional ultrasound have found success in preclinical microvessel research with mice models, the need for skull windows limits their current applicability in humans [15, 83].

### 4.1. Imaging (Direct)

With superior spatial and temporal resolution (Table 3), cerebral angiography remains the gold standard for imaging neurovascular diseases. Modern cerebral angiography uses digital subtraction angiography (DSA), where a mask‐radiograph is subtracted from a radiograph of the subject after contrast administration, thereby enhancing vascular structures [82]. Advancements in x‐ray detector technology have improved resolution as well as safety (reduced x‐ray exposure) [84, 77]. New variations of DSA include three‐dimensional DSA (3D‐DSA), where the detector is rotated around the subject, and time‐resolved 3D‐DSA (4D‐DSA), which involves stitching multiple 3D‐DSA volumes in series. However, traditional DSA remains superior in spatial resolution (50 *μ*m) and has been used by some researchers to define cerebral no‐reflow [78, 85, 86, 77].

CT angiography is increasingly replacing DSA for many neurovascular imaging tasks due to superior safety and cost‐efficiency [87, 88], but its spatial resolution remains inferior [77, 79]. A recent review on microvascular imaging cited ultrahigh resolution CT visualizing 40 *μ*m‐wide cerebral microvessels [89, 90], but this level of resolution is limited to microCT (small CT systems used for small preclinical models, e.g., mice) which, for reasons of optics, cannot be simply replicated in CT systems for much larger humans [91]. Newly FDA‐approved ultrahigh resolution CT [92], using photon‐counting detectors, can achieve 150 *μ*m resolution and can significantly improve visualization of lenticulostriate arteries, which are important in cerebral no‐reflow assessment [93, 94].

Retinal photography is being investigated for MvASR because cerebral and retinal vessels share common embryological, anatomical, and physiological features; retinal vessels are also comparatively easily accessible [95, 96]. Retinal photography can achieve resolutions < 10 *μ*m [76], and was used in multiple prospective studies to demonstrate the link between retinal vessel diameter changes (vasoreactivity) with stroke [97–99]. Mechanistically, vasoreactivity is believed to represent autoregulatory function, and its lack is also a marker of chronic hypertensive damage [95, 97]. Beyond manual assessment of retinal photographs, automated fractal analysis of retinal microvasculature may become a more cost‐effective MvASR method [96].

### 4.2. Imaging (Indirect)

“Next‐generation” 7‐Tesla MRI can achieve 350 *μ*m resolution, missing out most microvessels, let alone their details [80]. However, as aforementioned, MRI is able to detect CSVD‐related MRI biomarkers, which are known stroke risk‐factors [23, 26, 27, 29]. Recently, an exploratory study by Aminuddin et al. used 3‐Tesla MRI and found a relationship between decreased fractal dimension of cerebral vessels and CSVD severity (hence stroke risk) [13]. Due to MRI′s sensitivity to tissue fluid content, it can be used to assess no‐reflow with MRI perfusion‐weighted imaging (PWI), a group of techniques that measure tissue perfusion with contrast [86].

Ultrahigh frequency ultrasound (> 95 MHz) can achieve < 30 *μ*m spatial resolution, but only at millimeter‐depth because attenuation increases with frequency, therefore relegating ultrasound as an indirect modality [100–102]. Doppler ultrasound, which exploits the Doppler effect caused by moving erythrocytes, can be used transcranially to assess CBF [103]. Transcranial Doppler (TCD) ultrasound exploits “acoustic windows” (corresponding to thin regions of the cranium) to view deep structures [104]. Microembolic “signals” can be detected with TCD, either by scrutinizing intense signals independent of the cardiac cycle, or Doppler power spectra [105, 106]. Multiple studies have linked microembolic signals to stroke risk [105, 107], but others have found the relationship to be nonsignificant [108, 109]. Mixed performance of TCD may be due to a large part of the population not having acoustic windows, and TCD does not allow assessment of microvessels in many cortical regions [104]. Ultrasound microvascular flow imaging (MVFI) is a recent advancement that uses proprietary algorithmic filters to enhance sensitivity for low‐velocity Doppler signals, for example, bloodflow in microvessels [110, 111]. MVFI may be applicable to TCD to better discriminate microvessel flow, but is similarly limited by cranial anatomy. Still, MVFI can help in MvASR by detecting microvascular neovascularization of peripheral atherosclerotic plaques, which is associated with plaque embolization and stroke [111, 81].

### 4.3. Nonimaging

CSVD is useful for developing nonimaging MvASR because of its long‐term progression, known clinical signs and wide neuro‐imaging literature [20]. Many monogenic CSVDs have been discovered, the most common being cerebral autosomal‐dominant arteriopathy with subcortical infarcts and leukoencephalopathy (CADASIL), which is rare in England (~5/100,000 people) [112]. Driven by mutations in the *NOTCH3* gene, CADASIL increases risk of ischemic stroke, is screened in preimplantation testing in countries like the United Kingdom, and genetic tests have high sensitivity and specificity (> 90%) [113–115]. Genome‐wide association testing (GWAS) of patients with WMH may yield useful tests of microvascular stroke risk, though existing GWASs tend to have low predictive value [116, 117].

Studies of post‐stroke ischemia may yield candidates for molecular MvASR. Investigators tended to search for brain parenchyma‐specific biomarkers [118], but microvessel‐related neutrophil stalling (see Section 3.2) led to studies of neutrophil/lymphocyte ratio (NLR) as a biomarker in post‐stroke patients [65, 119]. Although NLR changes post‐stroke are associated with mortality and poor function, they are not significantly associated with recurrent stroke [119].

## 5. Conclusion

In conclusion, the cerebral microcirculation is involved in stroke risk at multiple levels, from microvascular inflammatory changes following asymptomatic accumulation of microemboli, to the risk relationship with MRI biomarkers, to the inflammatory and no‐reflow phenomena following recanalization. These findings complement the traditional macrovessel‐centric paradigm of stroke risk and suggest that directly assessing microvascular health could improve stroke prediction and prevention. However, MvASR continues to be a relatively niche consideration in the clinic for various reasons, including heterogeneity of microvasculature, lack of therapies targeting microvascular dysfunction, and lack of studies showing clear clinical benefit [89]. Greater adoption will be likely when more MvASR modalities can directly visualize microvasculature. Promising technologies on the horizon include 11.7‐Tesla MRI, which may achieve < 50 *μ*m spatial resolution [120] and photoacoustic imaging. By exploiting the photoacoustic effect (matter emits sound waves upon absorption of light energy), photoacoustic imaging may achieve similar resolutions at centimeter‐depth through the skull, enough to visualize most microvessels [121, 122]. Although further studies demonstrating clinical benefit of MvASR are ultimately needed, the evidence reviewed here suggests that MvASR will become increasingly relevant to stroke prediction and prevention.

## Funding

No funding was received for this manuscript.

## Conflicts of Interest

The author declares no conflicts of interest.

## Data Availability

Data sharing is not applicable to this article as no datasets were generated or analyzed during the current study.
